# Empowering the Emission of Upconversion Nanoparticles for Precise Subcellular Imaging

**DOI:** 10.3390/nano11061541

**Published:** 2021-06-11

**Authors:** Iman Rostami

**Affiliations:** Laboratory of Biomolecular Research, Department of Biology and Chemistry, Paul Scherrer Institute, 5232 Villigen, Switzerland; iman.rostami@psi.ch or iman.rostami@dcb.unibe.ch

**Keywords:** upconversion nanoparticles, near-infrared, surface modification, organelle targeting

## Abstract

Upconversion nanoparticles (UCNPs) are a class of inorganic fluorophores that follow the anti-Stokes mechanism, to which the wavelength of emission is shorter than absorption. This unique optical behavior generates relatively long-lived intermediate energy levels of lanthanides that stabilize the excitation state in the fluorescence process. Longer-wavelength light sources, e.g., near-infrared (NIR), penetrate deeper into biological materials such as tissue and cells that provide a larger working space for cell biology applications and imaging, whereby UCNPs have recently gained increasing interest in medicine. In this report, the emission intensity of a gadolinium-based UCNP was screened by changing the concentrations of the constituents. The optimized condition was utilized as a luminescent nanoprobe for targeting the mitochondria as a distinguished subcellular organelle within differentiated neuroblastoma cells. The main goal of this study is to illustrate the targeting process within the cells in a native state using modified UCNPs. Confocal microscopy on the cells treated with the functionalized UCNPs indicated a selective accumulation of UCNPs after immunolabeling. To tackle the insolubility of as-synthesized particles in water-based media, the optimized UCNPs were surface-coated with polyamidoamine (PAMAM) dendrimers that due to peripheral amino groups are suitable for functionalizing with peptides and antibodies. Ultimately, we concluded that UCNPs are potentially versatile and ideal tools for NIR bioimaging and capable of making adequate contrast against biomaterials to be detectable in electron microscopy (EM) imaging.

## 1. Introduction

Nanomedicine is a developing field where materials science is partially integrated with biochemistry [1,2,3]. Improving the impact of theranostics and efficient therapeutics is the main goal of nanomedicine, and numerous novel treatments have already been reported to support the implementation of advanced nanomaterials in medicine [4,5]. The output of quick progress in the manufacturing and design of materials in the nanometer-scale has resulted in different types of nanoparticles (NPs) with different physical/chemical properties, shapes, and sizes. Lanthanide-doped NPs, so-called upconversion NPs (UCNPs), are a class of fluorophore NPs that convert near-infrared (NIR) wavelengths to visible (Vis) or ultraviolet (UV) wavelengths with the assistant of dopant activators or emitter elements in the core of nanocrystals [6,7,8]. Using UCNPs in bioimaging provides various advantages in comparison with other common fluorophores such as quantum dots (QDs), gold NPs, and organic dyes. Foremostly, low cytotoxicity with UCNPs has attracted the attention of biochemists to take advantage of the unique properties of these materials. If synthesizing and modifying methods are accomplished optimally, the surface of these nanocrystals might be well designed for the loading of bioprobes such as peptides and antibodies, as it has been always a concern in nanomedicine to provide better platforms for biolabeling [9]. In addition to the suitable surface of nanocrystals, the fluorescence efficiency of fluorophores is another crucial factor for manufacturing efficacious NPs. Improving the quantum yield (QY) of UCNPs remains one of the major considerations for scientists to enhance the efficiency of light microscopy in an aqueous environment. Recent studies have exemplified the intensity optimization of UCNPs for faster, easier, and more economical synthesizing methodologies [10,11].

Several elements can affect the QY of UCNPs, and regarding this, the core–shell strategy with the energy migration-mediated upconversion (EMU) mechanism has produced efficient and tunable upconversion luminescence (UCL) by employing migrators, such as gadolinium (Gd) and ytterbium (Yb) doped within sublattices [12,13]. Luminescence transition in core–shell UCNPs occurs between the nearest neighbors in the crystal lattice and optimal spectral overlaps [14]. Gd^3+^ and Yb^3+^, due to having large energy gaps, ^6^P_7/2_ − ^8^S_7/2_, 32 × 10^3^ cm^−1^, and ^2^F_5/2_ − ^2^F_7/2_, 10 × 10^3^ cm^−1^, respectively, might be supreme energy mediators to immigrate the energy between the layers of lanthanide-doped nanocrystals, which possess allowed absorption bands close to Gd^3+^- and Yb^3+^-exciting levels, such as Er^3+^, Tm^3+^, Eu^3+^, and Tb^3+^ [15,16,17]. Mai et al. showed that the concentration of activators is significantly effective at UCL, and it plays a major role in the quenching coefficient [18]. In our previous work, we showed that the concentration optimization of sublattice elements such as sensitizers within epitaxial layers can influence UCL intensity; therefore, rather than laser, UCNPs can be excited by LED lamps that do not irradiate a focused light beam, and this might ease wide-range in vitro and in vivo imaging [19]. Studies in previous decades have indicated that the concentration of host lattices either in core or in shell influence the crystal lattice structure and consequently the luminescence property [20]. However, the electron transmission in the core plays a key role in the luminescence behavior with NIR-responsive NPs. Optimization in the structure and precursors of the crystal lattice is one of the main factors for the manufacturing of UCNPs with high QY luminescence [21,22].

Aside from having an appropriate crystal structure and potent UCL, concern has been expressed about the water solubility and biocompatibility of the biological application of UCNPs [23]. To this end, several techniques, utilizing epitaxial and nonepitaxial coatings, have been developed for improving the implementation of NPs in biomedical applications [24]. Appropriate surface modification of NPs is essential for accurate and efficient targeting. NPs larger than 20 nm in diameter are capable of being loaded by the most common types of bioprobes such as antibodies and nucleic acids, and intriguingly, NPs smaller than 100 nm are capable of passing through cell membranes by different pathways without rigid prevention [25]. Numerous studies have shown that specific protein targeting plays a prominent role in reducing the risk of elimination by the innate immune system and boosting cellular uptakes [26].

Cellular imaging has been remarkably promoted by integrating electron microscopy (EM) as an indispensable tool to the field. The wavelength of an electron at 100, 200, and 300 keV in electron microscopes is 3.70, 2.51, and 1.96 pm, respectively, which makes EM the most powerful technique with the highest resolution in imaging mode in comparison to the other available methods such as light and X-ray microscopies [27]. The development of three-dimensional (3D) structure information of tissues, cells, and organelles using cryo-electron tomography has been reached to a few nanometers, and this evolution might be promotive in cellular imaging for characterization of the explicit structure of biological samples [28,29,30].

## 2. Materials and Methods

### 2.1. Synthesizing of Core–Shell UCNPs

**Figure 1:**
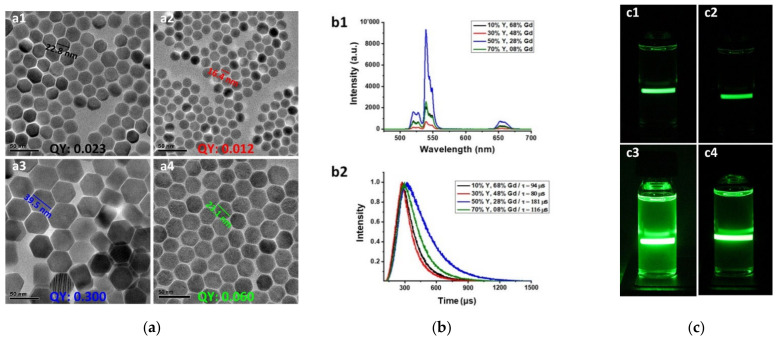
(**a**) TEM micrographs of as-synthesized UCNPs composed of different ratios of identical precursors of various sizes; pertinent sizes and QYs are noted. Room-temperature (**b1**) upconversion emission spectra and (**b2**) lifetime of core–shell β-NaGd_α1_Y_β_F_4_:Yb,Er@NaGd_α2_Y_β_F_4_:Yb@NaGd_α3_Y_β_F_4_:Yb NPs (black: α1, 2, and 3, respectively = 68, 70, and 70%/β = 10%; red: α1, 2, and 3, respectively = 48, 50, and 50%/β = 30%; blue: α1, 2, and 3, respectively = 28, 30, and 30%/β = 50%; green: α1, 2, and 3, respectively = 8, 10, and 10%/β = 70%; 20 mg/mL) under excitation of 980 nm; (**c**) photographs of as-synthesized UCNPs composed of different ratios of identical precursors that produce various fluorescence intensities against a 980 nm CW laser. Scale bars: 50 nm.

Boosting the luminescence efficiency and the QY of fluorophore NPs is one of the main concerns for researchers, especially in deep-tissue light imaging. Several studies have reported the luminescence size dependency of UCNPs [23,31], according to which, Xue et al. indicated that the quantum efficiency of particles (LiYF_4_:Yb^3+^,Er^3+^) decreases with the reduction of size from microscale to nanoscale, which is mainly due to higher surface-quenching effects [32]. Equal efforts have been paid to reduce nonradiative decay rates of UCL by tuning the crystal lattice of UCNPs [33], toward which Xu et al. designed a systematic optimization approach by doping different doses of host lattices to optimize the size, morphology, and UCL of KLu_2_F_7_:Yb^3+^,Er^3+^ UCNPs [34]. Herein, a facile thermal decomposition-based method is reported for enhancing the UCL intensity of core–shell β-NaGdYF_4_:Yb,Er@NaGdYF_4_,Yb@NaGdYF_4_:Yb NPs (Figure S2a). Details of the protocol are described in the Supplementary Materials. In this protocol, by adjusting the ratio of two host lattices (Y^3+^ and Gd^3+^), different sizes of grain NPs were obtained, and subsequently, the emission efficiency was affected. Based on our previous study, the Yb^3+^ concentration was optimally fixed at 20% of the mole in each layer, and according to that, the concentration of Y^3+^ and Gd^3+^ was proportionally altered [19]. Four different rational concentrations were examined, and the result was four different sizes of hexagonal-phase NaGdYF_4_-based nanocrystals (Figure 1a). Wide field-of-view transmission electron microscopy (TEM) of as-synthesized UCNPs indicated high homogeneity in the size of each batch. High-magnification TEM of a single nanocrystal indicated heterogeneity in the orientation of crystal planes, and the investigation of this phenomenon is beyond the scope of this paper (Figure S2b).

### 2.2. Functionalization of the UCNPs

In a classic chemical experiment, 50 mg of oleate-capped UCNPs (Figure S3a) dispersed in cyclohexane/ethanol (1:2) solution were mixed with 20 mL of 0.1 M HCl solution and stirred for 2 h. After that, the aqueous solution was centrifuged to pellet the UCNPs and separate them from the supernatant. To avoid wasting the NPs, the pellet was washed two times with 1× PBS and dispersed by pipetting in an ultrasonic water bath. The resulting ligand-free UCNPs (Figure S3b) were incubated with 4 mL of thioglycolic acid/water (1/2) and stirred overnight. The resulting carboxyl-terminated UCNPs were activated by EDC (100 mg) and NHS (20 mg) in 5 mL of water for 1 h and then mixed with 4 mL of PAMAM/water (5 mg/mL) solution for overnight stirring. The resulting PAMAM-NH2-modified UCNPs (Figure S3c) were centrifuged and washed 3 times to remove unconjugated PAMAMs and reacting reagents. Amino groups can react and conjugate with the carbonyl group of the antibodies (hard chain) and carbon domain of peptides. The PAMAM-modified UCNPs and 1 mg of peptide Pep-1 were stirred along with HATU/DIPEA in 4 mL of aqueous solution for 1 h. The resulting peptide-conjugated UCNPs were centrifuged at 10,000 rpm and washed to be prepared for conjugation with antibodies. The formed peptide-labeled UCNPs were dispersed in 0.5 mL of PSB1 and 5 µL of VDAC-1 antibody along with EDC (10 mg) and NHS (2 mg) in 0.5 mL Eppendorf tubes and incubated in an Eppendorf thermomixer with a 300 rpm speed at 18 °C for 2 h. The antibody-peptide-labeled UCNPs with high dispersion in aqueous solution (Figure S3d) were diluted and suspended in 1× PBS and stored at 4 °C until the moment of use.

### 2.3. Cell Culture

Differentiation of the SH-SY5Y human neuroblastoma cell line was accomplished within quartz-bottom dishes. This cell line allows us to provide an appropriate in vitro system to study the translational models for human disease, especially in neurobiology; however, the main purpose of using differentiated cells in this work was to get thin extensions for cryo-EM analysis. Culturing and differentiation of SH-SY5Y cells were performed by following the protocol of [35]. Briefly, one aliquot of the frozen SH-SY5Y neuroblastoma cells was rapidly thawed at 37 °C in a water bath, and after removing the old media by centrifugation, the cells were plated onto a T-25 flask. The cells were incubated at 37 °C, 5% CO_2_, to reach the confluence of 75–85% in basic grow media. The healthy cells were afterward detached from the T-25 flask using warmed 0.05% Trypsin-EDTA and seeded onto 35 mm quartz-bottom Petri dishes, which were coated with poly-D-lysine with a population density of 50 K cells/mL. The procedure took 18 days in total, and 3 differentiating media were required that contained retinoic acid as the key factor for differentiating. Figure S9a shows the unmatured differentiated cells at Day 1, and Figure S9b shows the matured cells at Day 18 with long and thin elongated neuritic extensions.

### 2.4. Differentiation of SH-SY5Y Cells on Gold-Coated TEM Grids

Cryo-EM on frozen cells was performed by the FEI Tecnai Spirit (120 kV) electron microscope. The cell culturing and differentiation procedure was performed as described above with one additional grid preparation step onto the Petri dish prior to the cell culture. As shown in Figure S11a, gold-coated Quantifoil Holey Carbon 2/2 TEM grids were glow-discharged for 10 s and placed onto coverslip-bottom Petri dishes, facing carbon-side up, and coated with poly-D-lysine for 1 h. The poly-D-lysine was rinsed gently using dd-H_2_O without disturbing the grid and dried under the cell culture hood after aspirating the dd-H_2_O. The grids with intact integrity, which were adequately adhered to the bottom of the Petri dish, were employed for culturing the cells. It should be noticed that a tiny gap between the grid and the Petri dish might lead the cells to move below the grid, as the cells prefer to sit on the top of the poly-lysine-coated glass rather than the grid. The SH-SY5Y cells after differentiation and treatment with UCNPs were physically fixed by freezing in liquid ethane that preserved enough cold temperature by liquid nitrogen while the fixation procedure was running. Vitrobot is a plunge freezer that is widely used for cryo-EM studies. By setting the plunging number to zero and manual blotting from the backside of the grid (reverse blotting), we were able to remove the extra solution from the grid. The grids with the mature differentiated cells on top (Figure S11b) needed to be rinsed with adequate pure water before freezing to remove all the salts and extra proteins from the cell culture media. Once the frozen-hydrated grid was prepared, it was inserted in the cryo-holder of the electron microscope and kept at approximately −180 °C throughout the experiment.

### 2.5. Cell Imaging by Confocal Microscopy

Differentiated SH-SY5Y cells were treated with functionalized UCNPs with an anti-VDAC-1 antibody to target the membrane protein on the surface of mitochondria. The modified UCNPs were dispersed in 1× PBS and mixed with the cell media with a final concentration of 5 µg/mL. Staining was performed 3 times, each time after 1 h, followed by rinsing the cell media to ensure the adequate penetration of UCNPs into the cells and no excess particles remaining in the matrix. Adding a low concentration and timewise NPs along with proper washing prohibits overloading and clumping of NPs on the cell membrane and all over the Petri dish. Serial imaging of the treated cells along the *Z*-axis assists with manifesting different heights of the cell body and extensions that bear accumulated UCNPs.

## 3. Results and Discussion

The UCL intensity of the Gd^3+^-sensitized UCNPs is significantly affected by the Yb^3+^ ions, which enable excitation energy migration from sensitizer to activator. UCL efficiency, QY, and the lifetime could reach the maximum at the concentration of 50% Y^3+^ and 28% Gd^3+^ in the core and 50% Y^3+^ and 30% Gd^3+^ within the shells (Figure 1b). The UC spectroscopy measurements (steady-state emission, lifetime, and QY) were accomplished by an Edinburgh Automated FLS980 steady-state and time-resolved fluorescence spectrometer under excitation of 980 nm and a power density of 25 W cm^−2^ for QY. The photographs of the as-synthesized UCNPs resolved in cyclohexane under irradiation of a continuous wavelength (CW) laser also obviously indicate that the emission of larger UCNPs is remarkably higher (Figure 1c).

**Figure 2:**
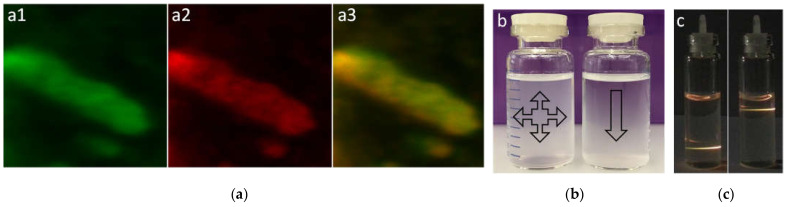
(**a**) Confocal imaging of as-synthesized β-UCNPs under 980 nm excitation by Multiphoton Leica SP8 equipped with NIR laser (**a1**) green and (**a2**) red channels; (**a3**) the merge of two channels. (**b**) Photography of surface-polished with HCl (right bottle) and PAMAM surface-modified (left bottle) UCNPs dispersed in water. Precipitation of UCNPs without PAMAM started relatively fast after suspension but PAMAM might keep the solubility of UCNPs up to a few days depending on the level of coated polymer around the NPs. (**c**) Photography of excited UCNPs-PAMAM by a 980 nm laser pen after suspension in water that shows high water-solubility and homogenous dispersity of UCNPs. The impact of hydroxyl groups from water and organic polymer coat around the particles influenced the luminescence of UCNPs by decreasing the ratio of green against red emission.

As-synthesized UCNPs were coated with oleic acid (OA), which is not water soluble and difficult to bind to biological materials; consequently, it was substituted with an appropriate water-soluble linker with chemically active groups (—NH_2_) at the terminal sides for binding to the C-domain (—COOH) of antibodies and peptides. Polyethylene glycol (PEG) with different molecular weights has been used very frequently for the surface modification of inorganic and organic NPs, but as a result of our empirical trials, PEG could not provide adequate solubility in aqueous solutions for our NPs with larger sizes in typical cell media and buffers such as 1× phosphate-buffered saline (PBS) and higher concentrations. Furthermore, this linker at high parenteral doses, especially higher molecular weights, shows certain toxicological effects and accumulation in organs such as the kidney [36]. These consequences compelled us to design a nonepitaxial shell for nanocrystals to boost their water solubility. Various techniques have been employed to transform hydrophobic rare-earth NPs to hydrophilic such as ligand exchange, ligand-free, and ligand interaction. Zhong et al. performed the surface modification of rare-earth NPs with a hydrophilic polymer shell by applying a van der Waals interaction between the alkyl chains of poly (maleic anhydride-alt-1-octadecene) and the oleic acid molecules on the UCNPs [19]. Herein, polyamidoamine (PAMAM) was employed as a water-soluble polymer with low toxicity to coat the UCNPs. Using PAMAM as a nonepitaxial shell around UCNPs provides the NPs the capability for multivalent conjugation, drug encapsulation, and bearing the organic dyes for the purpose of fluorescence resonance energy transfer (FRET) in combination with the UCL [37,38,39]. The OA on the surface of UCNPs was successfully replaced with the PAMAM generation 4 (G4) with 64 peripheral amino groups and a size of about 4 nm in diameter. The TEM micrographs after PAMAM modification show a lower electron density layer around the UCNPs, and the size is also compatible with the diameter of PAMAM G4 (Figure S3c). Visual observation using a laser pen, with a wavelength of 980 nm, revealed that there was no detectable agglomeration of UCNPs even in high-salt solutions after PAMAM coating, and these particles were homogeneously dispersed in water and 1× PBS (Figure 2).

The emission of modified UCNPs using laser pen and confocal microscopy indicated that the quenching factor and increment ratio of red to green emission is unavoidable after surface ligand exchange and removing the OA from the UCNPs’ periphery. Fluorescence decay caused by hydroxyl groups is a known defect on the emission of lanthanide ions-doped NPs and mainly influenced by electron transmission of the activator (Er^3+^, ^2^H_11/2_, ^4^S_3/2_ -> ^4^F_9/2_) and sensitizer (Yb^3+^, ^2^F_5/2_ -> ^2^F_7/2_) that could be the case for the core–shell types [40,41]. FTIR analysis also indicated the presence of fundamental groups in PAMAM and thioglycolic acid as the linker used for surface modification (Figure S4). PAMAM is positively charged due to a high population of amino groups located in the external layer, and the zeta potential measurement showed discernible change variation before and after UCNP modification (Figure S5). Positive charge associates with slowing the aggregation of NPs, and more importantly, according to the literature, it can reduce the value of transepithelial electrical resistance. which means the tight junctions between the epithelial cells can be loosed and the paracellular permeability increased [42,43]. Energy dispersive X-ray (EDX) analysis (Figure S6) of surface-modified UCNPs was performed on top of a gold TEM grid, and the results indicated the excessive condensation of oxygen and carbon from the polymeric shell in comparison with the diffusion rate of substances doped into the nanocrystals such as F, Yb, and Y. The amino groups from the PAMAM may cause an amide reaction with the carbonyl group in the carbon domain of peptides and/or antibodies, and the entire procedure of these covalent bondings provides reliable nanoprobes for targeting the biological objects (Figure S7).

Resazurin assay was performed for cell viability measurement. This analysis is based on the reduction of oxidized blue dye with a slight fluorescence to a pink fluorescent produced by living cells. The assay might be monitored by absorbance due to a minor blue shift with the visible light absorbance of the dye. The reduction of resazurin might be caused by reductive enzymes derived from mitochondria and cytosol [44]. The timewise (2, 8, 24 h) resazurin assay from UCNPs with three different coating shells in three individual concentrations (10, 50, and 100 µg/mL) indicated fainted cytotoxicity against the SH-SY5Y cell line (Figure S8).

**Figure 3:**
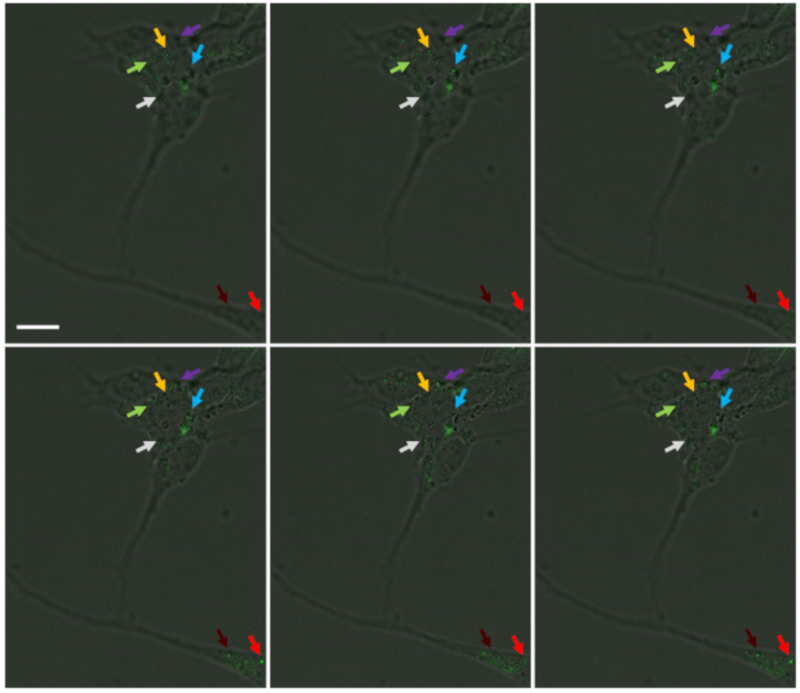
Confocal microscopy of the differentiated SH-SY5Y cells immunostained by UCNPs modified with Pep-1 and VDAC-1 antibody to target mitochondria. Z-stack serial imaging by a multiphoton microscope (Leica TCS SP8 MP) under excitation of 980 nm indicated the presence and accumulation of UCNPs in specific locations in both cell body and extensions. The same regions are marked with different arrow colors for better observation. Scale bar: 6 µm.

One of the main purposes of using the SH-SY5Y cell line was to have a differentiated structure of this cell line where it is possible to get thin extensions of the cells (Figure S9) for performing cryo-EM, as the regions with thick ices or nonvitrified areas are imperceptible by 120 kV electron microscopes. This study shows that the modified UCNPs are capable of penetrating into the cells and accumulating in certain locations within the cell body using Z-stack serial imaging by confocal microscopy (Figure 3). Pep-1 with both hydrophilic and hydrophobic domains assists in the better penetration of NPs into the cells [45]. The advantages of labeling the living cells assist in understanding the dynamic process and intracellular interactions and provide more relevant information compared to fixed-cell imaging. The SH-SY5Y cells after differentiation were treated with antibody-modified UCNPs for targeting mitochondria in living cells. The cells were treated without chemical fixation to visualize the near-native state of UCNP uptake by the cells, regardless of the exact mechanism for internalization, which is out of the scope of this study. The nonfixation approach might confirm the feasibility of the endocytosis procedure in living cells instead of creating artificial porousness on the cell membrane by fixative reagents. Innumerable studies have experimented with various strategies for targeting subcellular organelles with macromolecules and nanoparticles [46,47]. Among these approaches, confocal imaging has been heavily utilized for cellular imaging, but due to restrictions with light imaging, the resolution cannot be sufficient for visualizing the details of organelles in cells; therefore, we employed cryo-EM to elevate the resolution of microscopy from the deliberated mitochondria targeted by UCNPs. The differentiated SH-SY5Y cells were cultured on gold EM grids following the literature protocol by Shahmoradian et al., explained in Section 1 and Section 2, and plunged frozen in liquid ethane [48].

**Figure 4:**
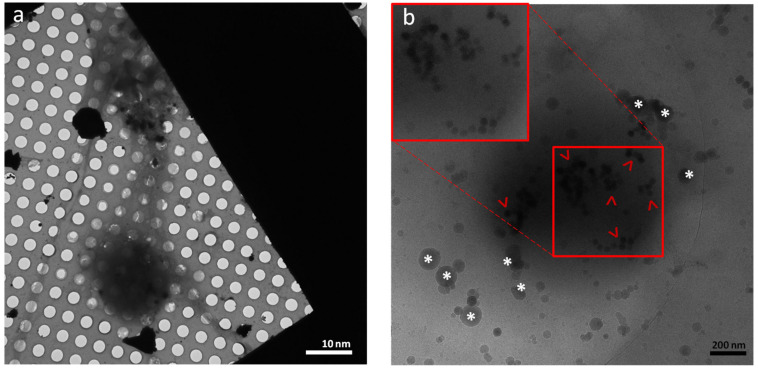
(**a**) Low-magnification cryo-EM image of vitrified differentiated SH-SH5Y cells with dendrite-like extensions on top of the TEM grid; (**b**) surface of a dense organelle that is coated with peptide/antibody-modified UCNPs (red arrowheads). Inserted red boxes show a relatively higher magnification and contrast of UCNPs. Ice crystals are labeled with white asterisks.

Frozen SH-SY5Y cells on the grid were transferred to a 120 kV electron microscope while well-preserved at cryogenic temperature, and imaging was performed (Figure 4a,b) in low-dose mode.

Initial screening showed well-vitrified differentiated cells on the surface of EM grids with adequate thin regions and extensions suitable for cryo-EM imaging (Figure 4a). The functionalized NPs were targeted specific organelles with the least amount of agglomeration. Figure 4b displays the surface of a dense organelle that is covered with modified UCNPs. At this stage, we are not capable to surely claim the targeted organelle is mitochondria as this requires the correlation of light and electron microscopy (CLEM) at cryogenic temperature, which we will accomplish in our future studies.

## 4. Conclusions

In summary, a core-shell approach was applied to design a bright fluorescent probe with a 540 nm emission under 980 nm excitation. The rationale for designing such composite UCNPs lies in the unique and potent optical properties of these materials for biological applications. It is also encouraging that the modified UCNPs caused no obvious in vitro toxicity to the mammalian cells, which is unlike typical inorganic dyes such as QDs. The surfaces of UCNPs were successfully modified with antibodies for targeting the mitochondria, and accordingly, this method can be applied for any subcellular organelle or pathogenic inclusions such as neurodegenerative hallmarks within the neurons and cells. The implementation of cryo-EM in 2D followed by fluorescence imaging in 2D widefield and 3D confocal modes provides a delicate technique for biological screening and localization of fluorescent NPs within cells. As the next step, the cryo-CLEM technique might be applicable, as a powerful cell imaging technique, to provide necessary localization information with light microscopy, merged with cryo-EM to obtain an adequate resolution for the detection of UCNPs. Cryo-electron tomography also may show a 3D view of organelles targeted with NPs that will be considered for future experiments.

## Data Availability

Data is contained within the article or supplementary materials.

## Supplementary Materials

The following are available online at https://www.mdpi.com/article/10.3390/nano11061541/s1, Figure S1: TEM micrographs of as-synthesized nanocrystals at core level, Figure S2: Atomic structure illustration of as-synthesized β-NaGdYF4:Yb,Er@NaGdYF4,Yb@NaGdYF4:Yb NP, Figure S3: TEMs of as-synthesized β-NaGdYF4:Yb,Er@NaGdYF4,Yb@NaGdYF4:Yb NPs, Figure S4: FTIR analysis of synthesized UCNPs coated with PAMAM, Figure S5: Zeta potential of NPs after before and after surface modification with PAMAM, Figure S6: EDX analysis of functionalized UCNPs, Figure S7: Schematic illustration of chemistry procedure for the fictionalization of the UCNPs with antibody and peptide, Figure S8: Time-dependent in vitro cytotoxicity analysis of functionalized UCNPs, Figure S9: Light microscopy of unmatured and matured differentiated SH-SY5Y cells, Figure S10: Schematic drawing of localization of mitochondria within the cell by functionalized UCNPs, Figure S11: Schematic procedure of TEM grid preparation for growing the cells.
