# Supplementary material for: Empowering the Emission of Upconversion Nanoparticles for Precise Subcellular Imaging
Source: Nanomaterials (Basel). 2021 Jun 11;11(6):1541. doi: 10.3390/nano11061541 (PMC8230588; doi:10.3390/nano11061541)
Supplement: Supplementary file 1 [file nanomaterials-11-01541-s001.zip › nanomaterials-1221237-supplementary.pdf]

Supplementary Materials
Empowering the Emission of Upconversion Nanoparticles for Precise Subcellular Imaging

Iman Rostami
Laboratory of Biomolecular Research, Department of Biology and Chemistry, Paul Scherrer Institute, Villigen, PSI 5232, Switzerland.

Materials. All reagents and solvents were purchased from commercial suppliers including rare-earth oxides (RE: Y, Yb, Gd, Er), CF_3COONa, cyclohexane, trifluoroacetic acid, 1-[Bis(dimethylamino)methylene]-1H-1,2,3-triazolo[4,5-b] pyridinium 3-oxide hexafluorophosphate (HATU), thioglycolic acid and N,N-Diisopropylethylamine (DIPEA), 1-ethyl-3-(3-dimethylaminopropyl)carbodiimide hydrochloride (EDC), N-hydroxysulfosuccinimide (NHS) and resazurin sodium salt were purchased from Sigma Aldrich and used as received. Oleic acid (OA; 90%), oleylamine (OM; 80%), 1-octadecene (ODE; 90%) were purchased from Sigma-Aldrich and used without further purification. RE(CF3COO)$_3$ were prepared by the literature method.[1] Peptide Pep-1 was purchased from GenScript and VDAC-1 antibody purchased from Abcam.

Synthesis of core-shell β-NaGdYF$_4$:Yb,Er@NaGdYF$_4$:Yb@NaGdYF$_4$:Yb nanocrystals. The β-NaGdYF4:Yb,Er@NaGdYF$_4$:Yb@NaGdYF$_4$:Yb nanoparticles were synthesized following the protocol of our previous work with slight changes.[2] Optimizing the percentage of the constitutive elements performed as following: in the cores, 10% Y^{3+} and 68% Gd^{3+}, 30% Y^{3+} and 48% Gd^{3+}, 50% Y^{3+} and 28% Gd^{3+}, 70% Y^{3+} and 8% Gd^{3+} plus 2% Er^{3+} were doped for each batch. In the shells the same percentage of elements as the cores were loaded but the quantity of activator (2%) was added to the amount of Gd^{3+} which means the percentage of Gd^{3+} in each layer was 70%, 50%, 30%, and 10%, respectively. Briefly, in a conventional synthetic procedure of α-$NaGd_{\alpha1}Y_{\beta}F_4$:Yb,Er, 1 mM of CF_3COONa and 1 mM $RE(CF_3COO)_3$ [RE: 98% Gd, Y, Yb, plus 2% Er] were added to a mixture of OA (10 mM), OM (10 mM), and ODE (20 mM) in a 3-necked flask at room temperature. The solution was degassed for 10 minutes with magnetic stirring then heated to 120 °C under vacuum for 20 minutes to remove moisture and oxygen, and then an optically transparent solution appeared. The solution was then heated up to 325 °C and maintained for 1 hour under argon circulation. After cooling down to room temperature, an excess volume of ethanol was added into the flask. The obtained mixture was centrifuged at 8000 rpm for 6 minutes, rinsed with ethanol three times, then dispersed in 2 mL of cyclohexane. The synthetic procedure of β-NaGdYF$_4$:Yb,Er was the same as the protocol utilized to synthesize the α-NaGdYF$_4$:Yb,Er nanocrystals, except that 0.5 mM CF_3COONa and α-NaGdYF$_4$:Yb,Er nanocrystals prepared were added to a mixture of OA (20 mM) and ODE (20 mM) in a 3-necked flask and maintained at 305 °C for 2 hour under argon circulation. The afforded nanocrystals were dispersed in 2 mL of cyclohexane for further coating. Images from the core nanocrystals are shown in Figure S1.

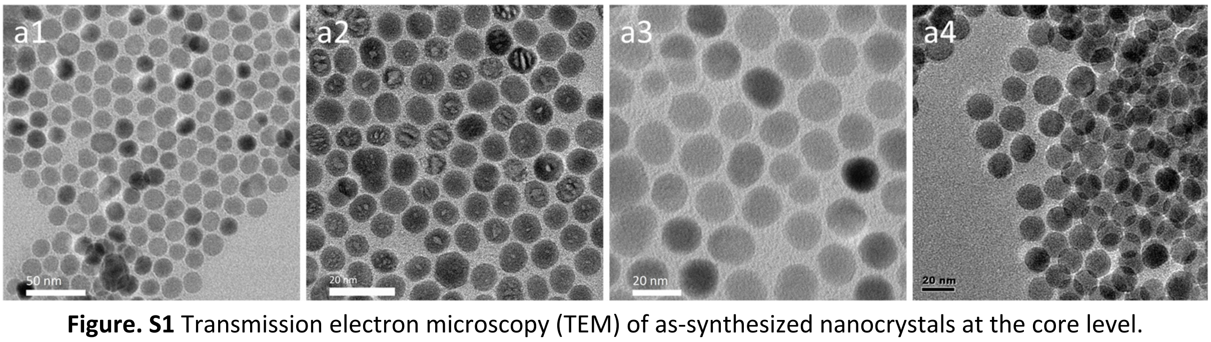

Figure. S1 Transmission electron microscopy (TEM) of as-synthesized nanocrystals at the core level.

For synthesizing of $NaGdYF_4$:Yb,Er@$NaGdYF_4$:Yb nanocrystals, 0.3 mM of CF_3COONa and 0.3 mM $RE(CF_3COO)_3$ [RE: 100% Gd, Y, Yb] plus the prepared β-$NaYF_4$:Yb,Er nanocrystals were added to a mixture of OA (20 mM), ODE (20 mM) in a three-necked flask at ambient temperature. The solution was degassed for 30 minutes with magnetic stirring then heated up to 120 °C under vacuum for 30 minutes to remove moisture and oxygen, and then an optically transparent solution should appear. The solution was then heated up to 305 °C for 1 hour and 310 °C for another 20 minutes under argon circulation. After cooling down to ambient temperature, an excess amount of ethanol was added into the flask. The resultant mixture was centrifuged at 8000 rpm for 6 minutes, rinsed with ethanol three times. The obtained nanocrystals were easily dispersed in cyclohexane.

For synthesizing of $NaGdYF_4$:Yb,Er@$NaGdYF_4$:Yb@$NaGdYF_4$:Yb nanocrystals. The synthetic procedure of $NaGdYF_4$:Yb,Er@$NaGdYF_4$:Yb@$NaGdYF_4$:Yb was the same as that used to synthesize $NaGdYF_4$:Yb,Er@$NaGdYF_4$:Yb nanocrystals, except that 1 mM CF_3COONa, 1 mM $RE(CF_3COO)_3$ [RE: 100% Gd, Y, Yb] and the $NaGdYF_4$:Yb,Er@$NaGdYF_4$:Yb nanocrystals prepared were added to a mixture of OA (20 mM) and ODE (20 mM) in a three-necked flask and maintained at 305 °C for 1 hour and 310 °C for another 20 minutes under argon circulation. The obtained nanocrystals were easily dispersed in cyclohexane.

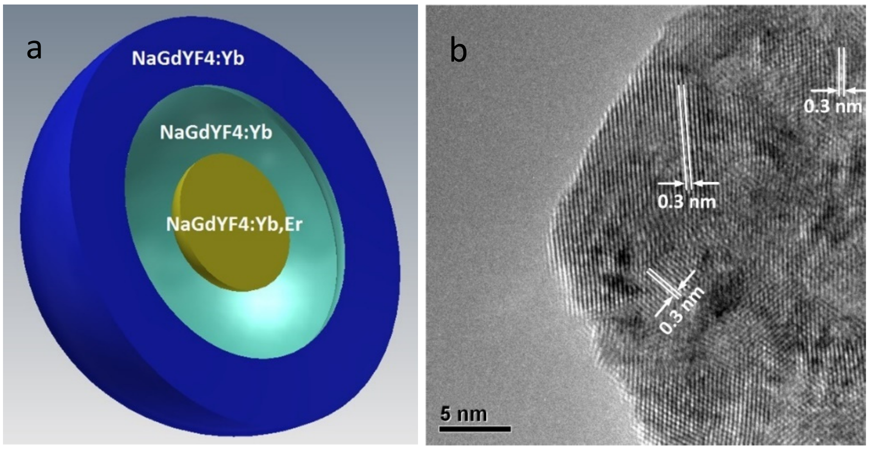

Figure. S2 (a) Schematic illustration of core-shell UCNP and the utilized elements. **(b)** High-magnification TEM micrograph of as-synthesized β-$NaGdYF_4$:Yb,Er@$NaGdYF_4$,Yb@$NaGdYF_4$:Yb NP by FEI, Tecnai F20.

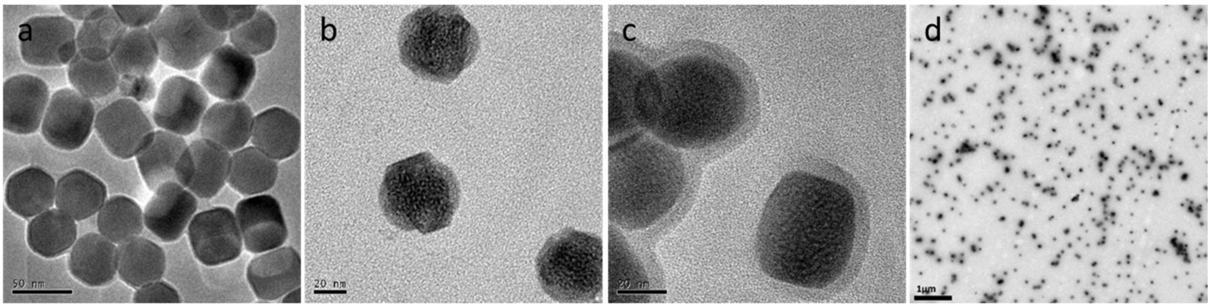

Figure. S3 TEMs of as-synthesized β-$NaGdYF_4$:Yb,Er@$NaGdYF_4$,Yb@$NaGdYF_4$:Yb NPs (**a**) as-synthesized, (**b**) surface-polished by HCl, (**c**) surface-modified with PAMAM and (**d**) low-magnification TEM imaging shows well-dispersity of UCNPs after surface-modified with PAMAM.

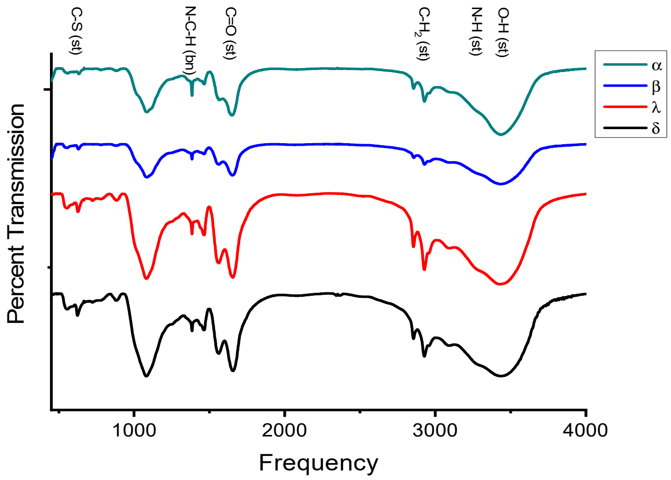

Figure. S4 FT-IR analysis of synthesized UCNPs coated with PAMAM. The stretch peak corresponding to carbonyl groups of PAMAM is appeared at around 1650 cm^{-1} due to shifting affect from adjacency of the amino group. Amino group peaks are hidden by hydroxyl stretch peaks that are unavoidable contamination. A weak stretch carbon sulfide peak that is corresponding to thioglycolic acid is appeared at 610 cm^{-1}.

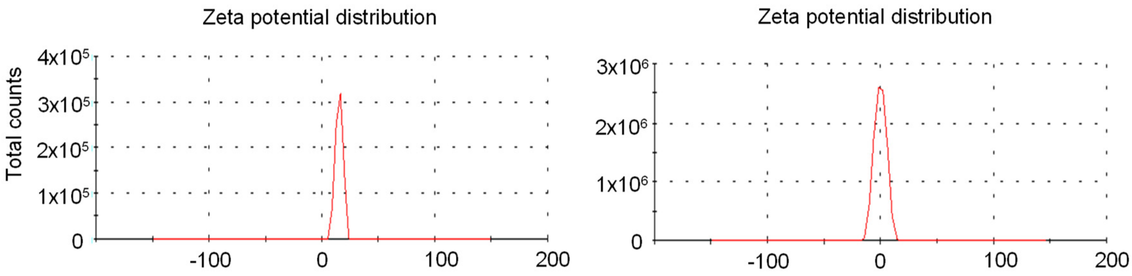

Figure. S5 Zeta potential of NPs after (left panel, 15.60 mV) and before (right panel, 0.019 mV) modification with PAMAM.

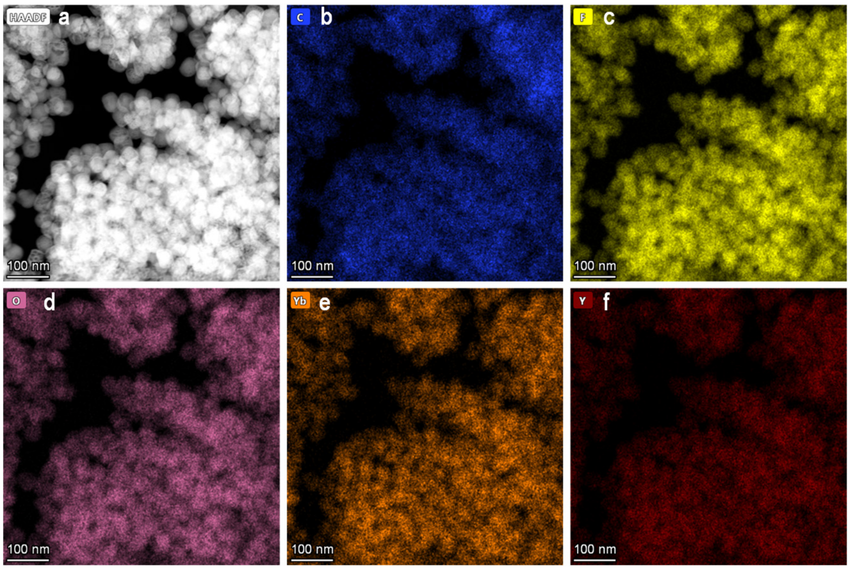

Figure. S6 (**a**) HAADF scanning transmission electron microscopy (STEM) image of functionalized UCNPs by FEI Talos F200X. The energy-dispersive X-ray (EDX) analysis elemental maps show the distribution of (**b**) carbon, (**c**) fluorine, (**d**) oxygen (**e**) ytterbium and (**f**) yttrium. To avoid interference of carbon film on typical TEM grids, the sample was loaded on top of a gold grid.

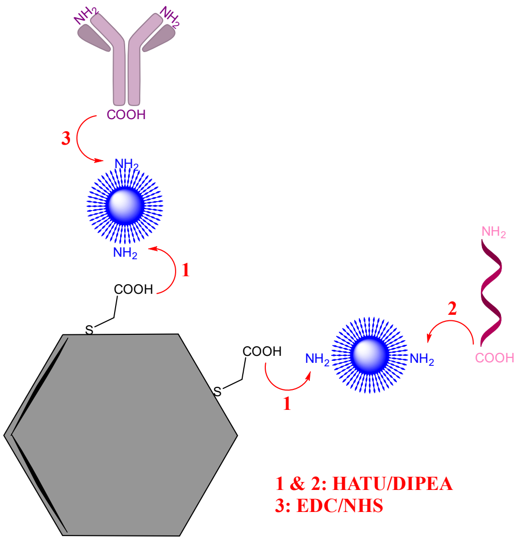

Figure. S7 Schematic illustration of chemistry procedure for the fictionalization of the UCNPs with antibody and peptide.

In-vitro light-controlled cytotoxicity. Cellular toxicity of various modified UCNPs was further investigated by Resazurin method. The Resazurin assay is based on the reduction of the oxidized blue dye with a slight fluorescence to a pink fluorescent produces by living cells. To describe the procedure briefly, high-purity Resazurin powder was dissolved in 1X-PBS (pH 7.4) to 0.15 mg/ml and filtered through a 0.22 µm sterile filter and stocked in 4 °C protected from light. SH-SY5Y cells were seeded for 36 hours in 96-well plates with confluency of 15K/well and a final volume of 100 µl/well. The cell media was refreshed with the media containing three different concentrations of modified UCNPs as mentioned above and the fluorescence abbreviation recorded by the PHERAstar FSX upon 575 nm excitation and 620 nm emission.

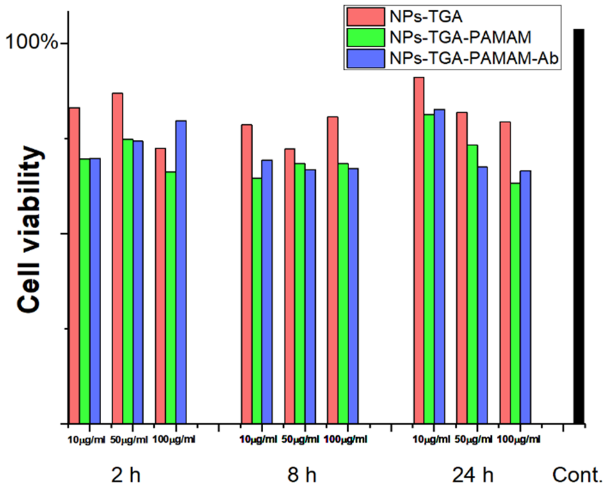

Figure. S8 Time-dependent in-vitro cytotoxicity by Resazurin assay of UCNPs coated with TGA (red) TGA-PAMAM (green) and TGA-PAMAM-antibody (blue) in three different concentrations (10, 50, and 100 µg/mL) against differentiated SH-SY5Y cells showed that there is no significant toxicity with modified UCNPs. Three independent assays were fulfilled in triplicate for each measurement.

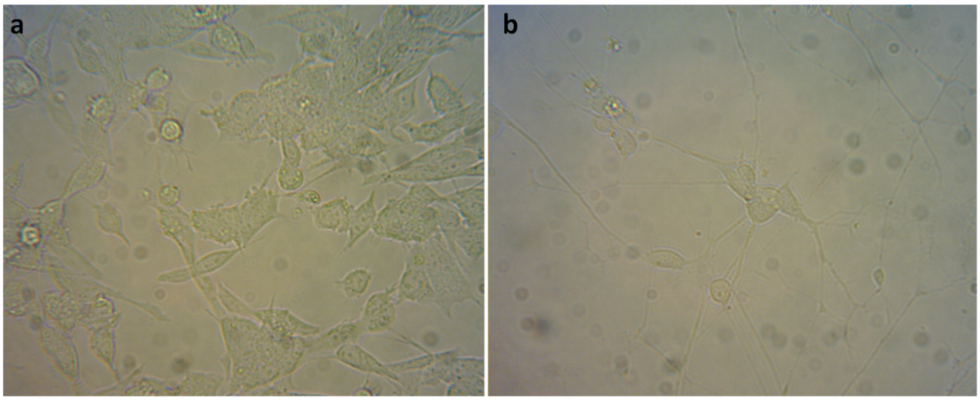

Figure. S9 (**a**) Morphological appearance of unmatured differentiated SH-SY5Y cells with the epithelial-like phenotype and (**b**) morphological appearance of differentiated SH-SY5Y cells with long neuritic extensions at 40X magnification using an inverted light microscope.

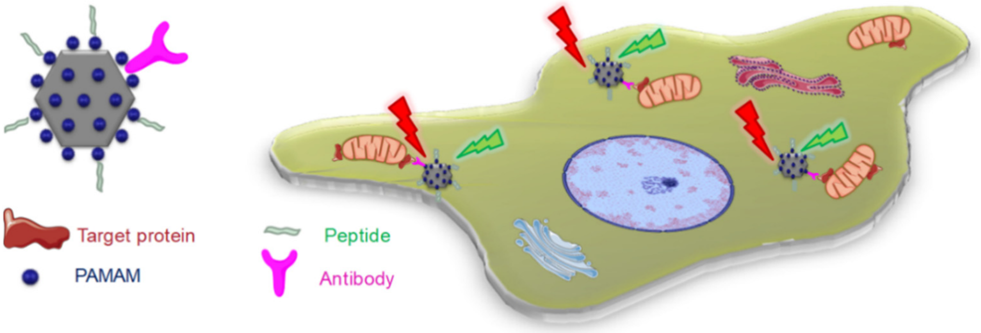

Figure. S10 Schematic drawing for localization of mitochondria within the cell by functionalized UCNPs for targeting of VDAC-1 protein under NIR light exposure.

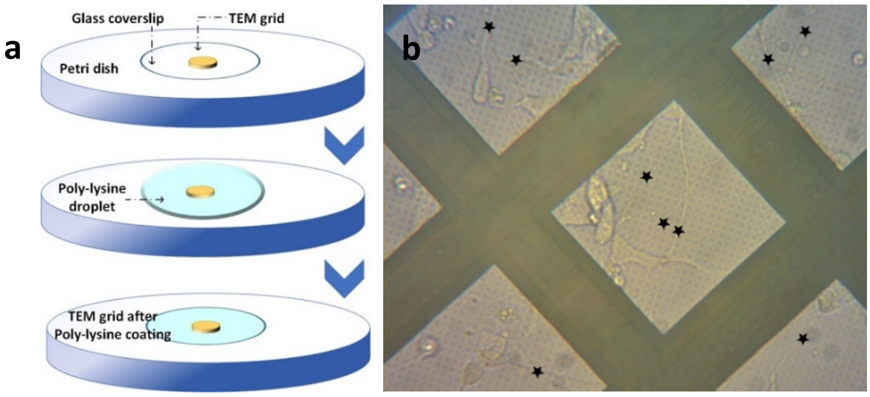

Figure. S11 (**a**) Schematic illustration of gold TEM grid preparation for growing the cells on the top. (**b**) The light micrographs of differentiated SH-SY5Y cells on the top of the gold TEM grid. The star marks indicate the long neuritic extensions at 40X magnification using an inverted light microscope.

References

1. Mai, H.-X.; Zhang, Y.-W.; Si, R.; Yan, Z.-G.; Sun, L.-D.; You, L.-P.; Yan, C.-H. High-Quality Sodium Rare-Earth Fluoride Nanocrystals: Controlled Synthesis and Optical Properties. *J. Am. Chem. Soc.* **2006**, *128*, 6426–6436, doi:10.1021/ja060212h.
2. Zhong, Y.; Rostami, I.; Wang, Z.; Dai, H.; Hu, Z. Energy Migration Engineering of Bright Rare-Earth Upconversion Nanoparticles for Excitation by Light-Emitting Diodes. *Adv. Mater.* **2015**, *27*, 6418–6422, doi:10.1002/adma.201502272.
